# 

**Published:** 1832-04-01

**Authors:** 


### CONTENTS

OF THE

### MEDICO-CHIRURGICAL REVIEW.

No. XXXII. APRIL 1, 1832.

### REVIEWS.

I.

Dr. Bright's Reports of Medical Cases, illustrating the Symptoms and Cure of Diseases *(Last Article)* .. 321

II.

A Treatise on the Diseases of the Heart and Great Vessels. By J. Hope, M.D. *(First Analytical Article)* .. 356

III.

Practical Observations on Prolapsus of the Rectum. By F. Salmon, Esq. .. 369

IV.

Elements of Practical Chemistry. By David Boswell Reid .. 374

V.

Abrégé Pratique des Maladies de la Peau, d'après les Leçons de Biett. Par Alphee Cazenave .. 382

VI.

Medico-Historical Account of the Western Coast of Africa. By James Boyle .. 408

VII.

Observations on Mental Derangement. By Andrew Combe, M.D. .. 423

VIII.

Treatise on Cholera Asphyxia. By G. H. Bell, Esq. *(Second Edition)* .. 434

IX.

Pamphlets on the Cholera .. 439

I. Letters on Cholera. By Whitelaw Ainslie, M.D. .. 439

II. Dr. Thackrah on the Cholera of Leeds .. 441

X.

The Cyclopædia of Practical Medicine. Parts I. and II. .. 445

XI.

A Treatise on the Anatomy of Regions. By Alf. Velpeau. Translated by Dr. Sterling .. 478

XII.

Etiology of the Reigning Epidemic .. 484

XIII.

Report of the Madras Medical Board on Epidemic Cholera. Extracted verbatim from the original document, drawn up by order of Government, 1824 .. 497

*(Continued in the Appendix, p. 609).*

### PERISCOPE.

1. Translations and Selections from Gregory and Celsus, and the Pharmacopœia
   - A. Mr. Leach's, 1828. .. .. .. .. .. .. .. .. .. .. .. .. .. } 513
   - B. Mr. Leach's, 1831. .. .. .. .. .. .. .. .. .. .. .. .. .. } 513
2. Cases of Iliac Aneurism successfully treated by operation. By Messrs. B. Cooper and Guthrie .. .. .. .. .. .. .. .. .. .. .. .. .. 514
3. Cases of Fungus Cerebri .. .. .. .. .. .. .. .. .. .. .. .. .. 518
4. Surgical Report from St. Thomas's Hospital
   - A. Aneurism of both Carotid Arteries (one tied) .. .. .. .. .. .. .. .. .. 520
   - B. Aneurism of the Popliteal Artery .. .. .. .. .. .. .. .. .. .. 524
   - C. Death after operation for Fungus of the Neck .. .. .. .. .. .. .. 525
5. Dr. Ogden (of Sunderland) on the Cholera .. .. .. .. .. .. .. .. .. 533
6. Dr. M'Robin on the Post-Mortem Appearances in cases of Insanity.. .. .. 538
7. The Cholera at Sunderland, Newcastle, and Gateshead
   - A. Dr. Adair Lawrie's Pamphlet .. .. .. .. .. .. .. .. .. .. .. } 541
   - B. Dr. David White's Pamphlet .. .. .. .. .. .. .. .. .. .. .. } 541
     - *a.* Contagion of Cholera .. .. .. .. .. .. .. .. .. .. .. .. 541
     - *b.* Appearance at Hillhead .. .. .. .. .. .. .. .. .. .. .. 542
     - *c.* Endemic characters .. .. .. .. .. .. .. .. .. .. .. .. 543
     - *d.* Cholera Hospitals.. .. .. .. .. .. .. .. .. .. .. .. .. 544
     - *e.* Quarantines .. .. .. .. .. .. .. .. .. .. .. .. .. .. 544
     - *f.* Sudden onset at Gateshead.. .. .. .. .. .. .. .. .. .. .. 545
     - *g.* Carried by the Wind .. .. .. .. .. .. .. .. .. .. .. .. 546
     - *h.* Susceptibility and Predisposition .. .. .. .. .. .. .. .. .. 547
     - *i.* Influence of Fear .. .. .. .. .. .. .. .. .. .. .. .. .. 547
     - *j.* Symptoms .. .. .. .. .. .. .. .. .. .. .. .. .. .. 548
     - *k.* Differences between the English and Indian .. .. .. .. .. .. 549
     - *l.* Treatment of the Stages .. .. .. .. .. .. .. .. .. .. .. 550
     - *m.* Short summary of the numbers attacked, comparative mortality, &c. 553
     - *n.* The Poor marked out as its victims .. .. .. .. .. .. .. .. .. 554
8. Dr. Weatherhead on the Beulah Spa .. .. .. .. .. .. .. .. .. .. 554
9. Cholera in Newcastle, Gateshead, Musselburgh, and Houghton-le-Spring. By Messrs. Greenhow, Moir, and Dodd
   - A. Mr. Greenhow's Work.. .. .. .. .. .. .. .. .. .. .. .. .. . 555
     - *a.* The progress of Cholera .. .. .. .. .. .. .. .. .. .. .. 556
     - *b.* Analysis of the symptoms .. .. .. .. .. .. .. .. .. .. .. 557
     - *c.* Treatment .. .. .. .. .. .. .. .. .. .. .. .. .. .. 558
     - *d.* The Causes .. .. .. .. .. .. .. .. .. .. .. .. .. .. 561
     - *e.* Acquired Predisposition .. .. .. .. .. .. .. .. .. .. .. 561
     - *f.* Fear and the Terrorists .. .. .. .. .. .. .. .. .. .. .. 562
     - *g.* Contagion .. .. .. .. .. .. .. .. .. .. .. .. .. .. 563
     - *h.* Atmospheric conditions .. .. .. .. .. .. .. .. .. .. .. 564
     - *i.* Prevalence of slight bowel-complaints .. .. .. .. .. .. .. .. 565
     - *j.* Postulates of Contagionists.. .. .. .. .. .. .. .. .. .. .. 566
     - *k.* Importation into Sunderland .. .. .. .. .. .. .. .. .. .. 566
     - *l.* Those assisting at Funerals, why attacked .. .. .. .. .. .. .. 568
     - *m.* Cholera in the Prison.. .. .. .. .. .. .. .. .. .. .. .. 569
     - *n.* Rivers and "the Great Channels" .. .. .. .. .. .. .. .. .. 570

o. Great mortality in Cholera Hospitals .. .. .. .. .. .. .. .. 571
B. Mr. Moir's Pamphlet .. .. .. .. .. .. .. .. .. .. .. .. .. .. 572
a. Contagiousness and religiousness of Scotland.. .. .. .. .. .. .. 572
b. The Musselburgh mortality.. .. .. .. .. .. .. .. .. .. .. .. 572
c. Cases at Musselburgh .. .. .. .. .. .. .. .. .. .. .. .. .. 573
d. Moonlight burials and fumigations .. .. .. .. .. .. .. .. .. .. 574
e. Strong evidence for contagion .. .. .. .. .. .. .. .. .. .. .. 575
f. Effects of Contagion graven on Tombs .. .. .. .. .. .. .. .. .. 576
C. Mr. Dodd on Cholera at Houghton-le-Spring .. .. .. .. .. .. .. .. 576
10. Nitric Acid in Tooth-ache. By Mr. Thomas .. .. .. .. .. .. .. .. 578
11. Principles of Obstetric Medicine. Parts II. and III. By David Davis, M. D.. 579
12. Dr. Fergusson and Mr. Brown on Cholera
A. Dr. Fergusson's Letters on Cholera .. .. .. .. .. .. .. .. .. .. 580
a. Contagion and "the Great Channels" .. .. .. .. .. .. .. .. 580
b. The Anti-contagionists .. .. .. .. .. .. .. .. .. .. .. .. .. 582
c. Moral courage, the best cordon.. .. .. .. .. .. .. .. .. .. .. 582
d. Effects of Contagion .. .. .. .. .. .. .. .. .. .. .. .. .. .. 583
B. Mr. Brown's Letter on Cholera at Musselburgh .. .. .. .. .. .. .. 584
a. Population, &c. of Musselburgh.. .. .. .. .. .. .. .. .. .. .. 585
b. Violence of Contagion at Musselburgh .. .. .. .. .. .. .. .. 585
c. Great public benefits from the Board at Musselburgh .. .. .. .. 586
d. Diary of a Contagionist .. .. .. .. .. .. .. .. .. .. .. .. 587
e. Immorality of Whiskey and morality of Wine.. .. .. .. .. .. 587
13. Mr. Hart on partial Fracture of the long bones in Children .. .. .. .. 588
Remarks on partial Fracture of the Fore-arm .. .. .. .. .. .. .. .. 590
14. Mr. Ferguson on the preparation of Soap Cerate .. .. .. .. .. .. .. 520
15. M. Cruveilhier on Acute and Chronic Laryngitis.. .. .. .. .. .. .. .. 591
Formation of matter external to the Larynx. Cases and remarks .. .. .. 592
16. Surgical Report by Mr. Laidlaw .. .. .. .. .. .. .. .. .. .. .. .. 594
A. Rupture of the Kidney .. .. .. .. .. .. .. .. .. .. .. .. .. .. 594
B. Suicidal wound of the Throat .. .. .. .. .. .. .. .. .. .. .. .. 596
17. Cyclopædia of Medicine. Part III.. .. .. .. .. .. .. .. .. .. .. .. 598
Drs. Quain and Adair Crawford on Inflammation of the Brain .. .. .. .. 598
Ulceration of the Brain .. .. .. .. .. .. .. .. .. .. .. .. .. .. .. 601
18. Dr. Stedman's Surgical Report.
A. Operation for Imperforate Vagina .. .. .. .. .. .. .. .. .. .. .. 602
B. Extirpation of Parotid Tumour, preceded by Ligature of the Carotid Artery 603
19. Mr. Clarke's important case of Cholera at Banbury .. .. .. .. .. .. .. 605
20. Dr. Yates's Letter on Cholera at Vienna .. .. .. .. .. .. .. .. .. .. 606
21. Dr. Seed's new remedy for Ophthalmia .. .. .. .. .. .. .. .. .. .. 607
22. Comparison between the Fever of Smyrna and the collapse stage of Cholera .. 607
23. Dr. O'Halloran's Letter to the Editor on the Epidemic Cholera .. .. .. 608

### APPENDIX.---EXTRA-LIMITES.

I.—Report of the Madras Medical Board on Epidemic Cholera. *(Continued from page 512.)* .. .. .. .. .. .. .. .. .. .. .. .. .. .. .. .. 609
Tabular Analysis of the individual Reports to the Board, respecting premonitory Diarrhœa and Consecutive Fever .. .. .. .. .. .. .. .. .. 622

Editorial remarks on this Report .. .. .. .. .. .. .. .. .. .. .. .. .. 627
Asphyxial stage.. .. .. .. .. .. .. .. .. .. .. .. .. .. .. .. .. 628
Consecutive fever .. .. .. .. .. .. .. .. .. .. .. .. .. .. .. .. 629
Sydenham's description of Cholera.. .. .. .. .. .. .. .. .. .. .. .. 630
Frank's description of Epidemic Cholera .. .. .. .. .. .. .. .. .. .. 631
II.—Mr. Cameron on the reigning Epidemic of London, as compared with that in India .. .. .. .. .. .. .. .. .. .. .. .. .. .. .. .. .. 633
III.—Mr. Dobson on the Epidemic Cholera at Leeds.. .. .. .. .. .. .. .. 636
IV.—Drs. Lorimer and Burton on the Cholera at Haddington.. .. .. .. .. .. 640
V.—History of the Progress of Cholera in England .. .. .. .. .. .. .. .. 645
*a.* Cholera at Sunderland .. .. .. .. .. .. .. .. .. .. .. .. .. 645
*b.* Statistics of that Towns .. .. .. .. .. .. .. .. .. .. .. .. 646
Its appearance at Newcastle .. .. .. .. .. .. .. .. .. .. .. .. 647
At North and South Shields .. .. .. .. .. .. .. .. .. .. .. .. 648
At Gateshead.. .. .. .. .. .. .. .. .. .. .. .. .. .. .. .. 649
At Haddington .. .. .. .. .. .. .. .. .. .. .. .. .. .. .. 650
At Tranent, &c. .. .. .. .. .. .. .. .. .. .. .. .. .. .. .. 651
At Preston Pans .. .. .. .. .. .. .. .. .. .. .. .. .. .. .. 652
Propagation by Contagion considered .. .. .. .. .. .. .. .. .. 653
Its Symptoms.. .. .. .. .. .. .. .. .. .. .. .. .. .. .. .. 655
Cases of various kinds .. .. .. .. .. .. .. .. .. .. .. .. .. 655
Cholera at the Mauritius .. .. .. .. .. .. .. .. .. .. .. .. .. 660
Post-Mortem appearances .. .. .. .. .. .. .. .. .. .. .. .. .. 660
Signs distinguishing it from common Cholera.. .. .. .. .. .. .. .. 662
Ratio Signorum .. .. .. .. .. .. .. .. .. .. .. .. .. .. .. 665
Treatment .. .. .. .. .. .. .. .. .. .. .. .. .. .. .. .. .. 669
Causes .. .. .. .. .. .. .. .. .. .. .. .. .. .. .. .. .. .. 672
VI. Dr. Yates on the Cholera at Ely, Cambridgeshire .. .. .. .. .. .. .. 674
VII. Concluding Observations on the Epidemic.. .. .. .. .. .. .. .. .. 675
VIII. Address to the Medical Profession.. .. .. .. .. .. .. .. .. .. .. 676
BIBLIOGRAPHICAL RECORD .. .. .. .. .. .. .. .. .. .. .. .. .. .. .. 677

